# Kinematics modeling of the gear-based crank mechanism engine regardless of the compressions ratio variations

**DOI:** 10.1038/s41598-024-53085-1

**Published:** 2024-02-02

**Authors:** Amir Sakhraoui, Maroua Saggar, Fayza Ayari, Rachid Nasri

**Affiliations:** 1 Department of Mechanical Engineering, LR-11-ES19 Applied Mechanics and Engineering Laboratory (LR-MAI), National Engineering School of Tunis, University of Tunis El Manar, 1002 Tunis, Tunisia; 2 Mechanical Laboratory of Sousse, Private Central Polytechnic School of Tunis, Centrale University, La Goulette, Tunis, Tunisia; 3 National Higher School of Engineers of Tunis (ENSIT), (99/UR/11-46), University of Tunis, 1002 Tunis, Tunisia

**Keywords:** Mechanical engineering, Engineering, Physics

## Abstract

In this work, geometric and kinematic modeling of the gear-based crank mechanism engine (GBCM) was performed. To this aim, a mechanical approach based on projective computational methods and mechanism theory laws is applied, to which a parametric study has allowed a conclusion on the geometrical and kinematic behaviors of the moving links. The study concluded that the kinematic quantities at connecting rod head are one-half of those at the piston top head. The extrinsic behavior like the stroke of the connecting rod head is twice the crank radius and the piston kinematic are identical to the conventional engine with the same crankshaft ratio regardless of the compression ratio and any gear wheel radius. Hence, all the extrinsic kinematic properties of a classic crankshaft mechanism of the fixed compression ratio engine remain valid for a gear-based crank mechanism engine and can be used for dynamic calculation purposes.

## Introduction

Environmental problems are making vehicle manufacturers lessen their fuel emissions by enhancing engine efficiency through optimized thermal control and growing the compression ratio. In this context, Variable compression ratio engines (VCR) make a contribution, in which the very last function of the Top dead center TDC piston is adjusted to optimize the compression ratio to increase thermodynamic performance, particularly at part load, and finally reduce gasoline consumption and CO2 emissions. Several VCR configurations presently exist^1–5^, such as the gear-based crank mechanism engine (GBCM) with variable compression ratio (VCR_MCE-5)^6–9^.

What makes the GBCM engine distinctive from the traditional constant compression ratio engine is the interposition of a managed equipment wheel between the piston and the crankshaft mechanism, which gives the advantage of a variable compression ratio.

In this work, we can talk about the results received via the GBCM engine. To evaluate the results of the proposed mechanism, we simulated kinematic parameters together with piston stroke, positions, velocities, and accelerations, which can be used for dynamic computational purposes followed in analytical computational codes for multi-body simulation (MBS)^10–13^.

The results confirm that kinematic parameters are obtained for the GBCM engine regardless of the value of the compression ratio i.e. regardless of the position of the control cylinder.

The aim is to kinematically simulate the VCR engine, the results of which are used to describe the kinematic behavior through a pre-established parameter setting.

**Figure 1:**
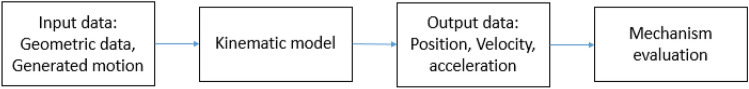
General bloc diagram to evaluate the kinematic behaviour.

Firstly, kinematic modeling and simulations were performed then dynamic analyses can be performed taking into account external forces. The analysis processes were carried out according to the general methodology described in Fig. 1. In the second section, we study the behavior laws of the VCR engine through assumptions and adequate parameterization to establish the solutions in terms of strokes, positions, speeds, and accelerations. Finally, we discuss the influence of the parameters on the kinematic behavior of the VCR engine to conclude the evolution of the kinematic steady-state quantities.

## Analytical study

**Figure 2:**
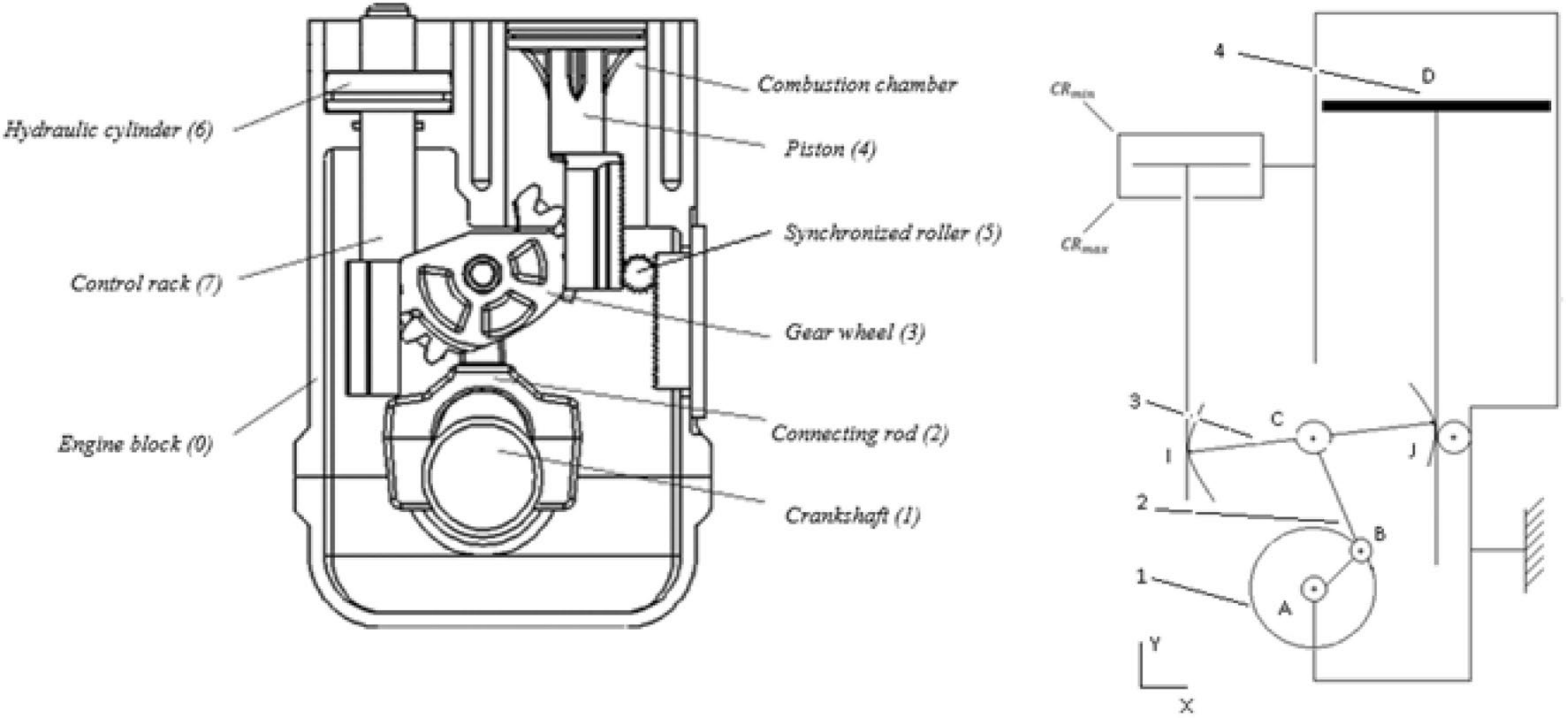
A representation of the GBCM engine structure.

Like in conventional engines, the crankshaft of the GBCM engine transforms an oscillating rectilinear displacement into a continuous rotation. The piston stroke between the TDC and BDC is not affected by the CR setting (Fig. 2). The hydraulic valve 6 and its control rack 7 are kinematically fixed so that have useful mobility equal to one.

A planar geometric parameterization $\left(A,{\varvec{x}}, {\varvec{y}},\boldsymbol{ }0\right)$ is used. The piston and the cylinder link are assumed to be a sliding link and the axis of the connecting rod head and the axis of the crank is concurrent: ${\varvec{y}}={\varvec{A}}{\varvec{C}}/\Vert {\varvec{A}}{\varvec{C}}\Vert$**.**

1 $${{\varvec{x}}}_{1}=\frac{{\varvec{A}}{\varvec{B}}}{\Vert {\varvec{A}}{\varvec{B}}\Vert }=\frac{{\varvec{A}}{\varvec{B}}}{{L}_{1 }}\boldsymbol{ }\boldsymbol{ },\boldsymbol{ }\boldsymbol{ }{\boldsymbol{ }{\varvec{x}}}_{2}=\frac{{\varvec{B}}{\varvec{C}}}{\Vert {\varvec{B}}{\varvec{C}}\Vert }=\frac{{\varvec{B}}{\varvec{C}}}{{L}_{2}}$$

2 $${\theta }_{1}=\left({{\varvec{x}}\widehat{,}{\varvec{x}}}_{1}\right) , {\theta }_{2}=\left({{\varvec{x}}\widehat{,}{\varvec{x}}}_{2}\right), {\theta }_{3}=\left({{\varvec{x}}\widehat{,}{\varvec{x}}}_{3}\right)$$

3 $${\varvec{x}}=\frac{{\varvec{C}}{\varvec{J}}}{\Vert {\varvec{C}}{\varvec{J}}\Vert }, {\varvec{y}}=\frac{{\varvec{A}}{\varvec{C}}}{\Vert {\varvec{A}}{\varvec{C}}\Vert }=\frac{{\varvec{J}}{\varvec{D}}}{\Vert {\varvec{J}}{\varvec{D}}\Vert }$$

In this section, a geometric closure connecting A-B-C-J-D-A points leads to the kinematic laws as follows:

4 $${\varvec{A}}{\varvec{D}}={\varvec{A}}{\varvec{B}}+{\varvec{B}}{\varvec{C}}+{\varvec{C}}{\varvec{D}}\boldsymbol{ }\Rightarrow \boldsymbol{ }{\varvec{A}}{\varvec{D}}={\varvec{A}}{\varvec{C}}+{\varvec{C}}{\varvec{J}}+{\varvec{J}}{\varvec{D}}$$

Input parameter ${y}_{3}^{C}$ denotes the translation of the gear wheel 3 in C.

Output parameter ${\theta }_{1}$ denotes the crankshaft rotation 1 in A.

With:

5 $${\varvec{A}}{\varvec{C}}={\varvec{A}}{\varvec{B}}+{\varvec{B}}{\varvec{C}}\iff {y}_{3}^{C} {\varvec{y}}={L}_{1}{\boldsymbol{ }{\varvec{x}}}_{1}+{L}_{2} {{\varvec{x}}}_{2}$$

By projection onto the global reference frame $R\left({\varvec{x}},{\varvec{y}}, {\varvec{z}}\right)$ we obtain:

6 $${{y}_{3}^{C}=L}_{1 }{\text{sin}}{\theta }_{1}+{L}_{2} \sqrt{1-{\left(\lambda {\text{cos}}{\theta }_{1}\right)}^{2}}={L}_{1}\left({\text{sin}}{\theta }_{1}+\sqrt{\frac{1}{{\lambda }^{2}}-{\left({\text{cos}}{\theta }_{1}\right)}^{2}}\right)$$

7 $$\left\{\begin{array}{c}AC={y}_{3}^{C} y\\ CJ+JD={R}_{3} x+{R}_{3}{\theta }_{3} y\end{array}\right.\Rightarrow {\varvec{A}}{\varvec{D}}={\left(\begin{array}{c}{x}_{4}^{D}\\ {y}_{4}^{D}\\ 0\end{array}\right)}_{R}={\left(\begin{array}{c}{R}_{3}\\ {y}_{3}^{C}+{R}_{3}{\theta }_{3}\\ 0\end{array}\right)}_{R}$$

### Determining GBCM velocity and acceleration fields

#### Geometric method

This approach is based on projection and derivation:

The piston velocity 4 is:

8 $$\frac{d {\varvec{A}}{\varvec{D}}}{dt}={\dot{y}}_{4}^{D} {\varvec{y}}= \left({\dot{y}}_{3}^{C}+{R}_{3}{\dot{\theta }}_{3}\right) {\varvec{y}}$$

With:

9 $${\varvec{A}}{\varvec{D}}={\varvec{A}}{\varvec{C}}+{\varvec{C}}{\varvec{J}}+{\varvec{J}}{\varvec{D}}$$

10 $${\dot{y}}_{4}^{D}={\dot{y}}_{3}^{C}+{R}_{3}{\dot{\theta }}_{3} \Rightarrow {\dot{y}}_{3}^{C}={\dot{y}}_{4}^{D}-{R}_{3}{\dot{\theta }}_{3}$$

The no-slip rolling condition of 3 with respect to 4 gives:

11 $${\dot{y}}_{4}^{D}=2 {R}_{3}{\dot{\theta }}_{3}$$

This involves:

12 $$\left\{\begin{array}{c}{\dot{y}}_{3}^{C}={\dot{y}}_{4}^{D}-{R}_{3}{\dot{\theta }}_{3}\\ {\dot{y}}_{4}^{D}=2 {R}_{3}{\dot{\theta }}_{3}\end{array}\right.\Rightarrow {\dot{y}}_{3}^{C}={R}_{3}{\dot{\theta }}_{3}$$

Finally, it is shown by time differentiation that the velocity and acceleration of piston 4 at D are two times greater than those calculated in the connecting rod head 2 at C:

13 $${{\dot{y}}_{4}^{D}=2 \dot{y}}_{3}^{C}=2 \frac{d }{dt}\left({y}_{3}^{C}\right)$$

14 $${\ddot{y}}_{4}^{D}=2 {\ddot{y}}_{3}^{C}=2 \frac{d }{dt}\left({\dot{y}}_{3}^{C}\right)$$

By derivation of (6) we obtain the speed as follows:

15 $${\dot{y}}_{4}^{D}=2 {L}_{1 }{\dot{\theta }}_{1}\mathrm{ cos}{\theta }_{1}\left[1+\frac{\lambda {\text{sin}}{\theta }_{1}}{\sqrt{1-{\left(\lambda {\text{cos}}{\theta }_{1}\right)}^{2}}}\right]$$

The acceleration ${\ddot{y}}_{4}^{D}$ in D of piston 4 is expressed as a function of the angular position ${\theta }_{1}$ and the rotational speed ${\dot{\theta }}_{1}$ of the crankshaft 1 in a steady state case by:

16 $${\ddot{y}}_{4}^{D}=2 {L}_{1} {{\dot{\theta }}_{1}}^{2} \left[\left(\frac{\lambda \sqrt{1-{\left(\lambda {\text{cos}}{\theta }_{1}\right)}^{2}}+\frac{{\lambda }^{3}{\left({\text{sin}}{\theta }_{1}\right)}^{2}}{\sqrt{1-{\left(\lambda {\text{cos}}{\theta }_{1}\right)}^{2}}}}{1-{\left(\lambda {\text{cos}}{\theta }_{1}\right)}^{2}}\right){\left({\text{cos}}{\theta }_{1}\right)}^{2}-\left(1+\frac{\lambda {\text{sin}}{\theta }_{1}}{\sqrt{1-{\left(\lambda {\text{cos}}{\theta }_{1}\right)}^{2}}}\right){\text{sin}}{\theta }_{1}\right]$$

#### Kinematic method

The kinematic approach employs the law of moments, the equi-projectivity of fields velocities, no-slip rolling, and the velocity motion composition between parts. Therefore, the relationship of velocities between the piston at D and the connecting rod head at C in relation to the structure's frame:

17 $${{\varvec{V}}}_{3}^{{\varvec{C}}}={{\varvec{V}}}_{3}^{{\varvec{I}}}+{\varvec{C}}{\varvec{I}}\wedge {{\varvec{\Omega}}}_{3}$$

The no-slip rolling condition in relation to the structure's frame:

18 $${{\varvec{V}}}_{3}^{{\varvec{I}}}=0$$

19 $${{\varvec{V}}}_{3}^{{\varvec{C}}}={-R}_{3 }{\varvec{x}}\wedge {\dot{\theta }}_{3 }{\varvec{z}}={R}_{3}{\dot{\theta }}_{3} {\varvec{y}}$$

20 $${{\varvec{V}}}_{3}^{{\varvec{J}}}={{\varvec{V}}}_{3}^{{\varvec{C}}}+{\varvec{J}}{\varvec{C}}\wedge {{\varvec{\Omega}}}_{3}={R}_{3}{\dot{\theta }}_{3} {\varvec{y}}-{R}_{3 }{\varvec{x}}\wedge {\dot{\theta }}_{3 }{\varvec{z}}=2 {R}_{3}{\dot{\theta }}_{3} {\varvec{y}}$$

21 $$\left\{\begin{array}{c}{{\varvec{V}}}_{3}^{{\varvec{C}}}={R}_{3}{\dot{\theta }}_{3} y\\ {{\varvec{V}}}_{3}^{{\varvec{J}}}=2 {R}_{3}{\dot{\theta }}_{3} y\end{array}\right.\Rightarrow {{\varvec{V}}}_{3}^{{\varvec{C}}}=\frac{1}{2} {{\varvec{V}}}_{3}^{{\varvec{J}}}$$

Yet we have:

22 $${{\varvec{V}}}_{4}^{{\varvec{J}}}={{\varvec{V}}}_{43}^{{\varvec{J}}}+{{\varvec{V}}}_{3}^{{\varvec{J}}}$$

The no-slip rolling condition of 3 with respect to 4 gives:

23 $${{\varvec{V}}}_{43}^{{\varvec{J}}}=0$$

24 $${{\varvec{V}}}_{4}^{{\varvec{J}}}={{\varvec{V}}}_{3}^{{\varvec{J}}}$$

25 $${{\varvec{V}}}_{4}^{{\varvec{J}}}={{\varvec{V}}}_{4}^{{\varvec{D}}}+{\varvec{J}}{\varvec{D}}\wedge {{\varvec{\Omega}}}_{4}={{\varvec{V}}}_{4}^{{\varvec{D}}}\boldsymbol{ }\boldsymbol{ }\boldsymbol{ }$$

26 $$\left\{\begin{array}{c}{{\varvec{V}}}_{4}^{{\varvec{J}}}={{\varvec{V}}}_{3}^{{\varvec{J}}}\\ {{\varvec{V}}}_{4}^{{\varvec{J}}}={{\varvec{V}}}_{4}^{{\varvec{D}}}\end{array}\right.\Rightarrow {{\varvec{V}}}_{3}^{{\varvec{J}}}={{\varvec{V}}}_{4}^{{\varvec{D}}}$$

It can be deduced that:

27 $$\left\{\begin{array}{c}{{\varvec{V}}}_{3}^{{\varvec{C}}}=\frac{1}{2} {{\varvec{V}}}_{3}^{{\varvec{J}}}\\ {{\varvec{V}}}_{3}^{{\varvec{J}}}={{\varvec{V}}}_{4}^{{\varvec{D}}}\end{array}\right.\Rightarrow {{\varvec{V}}}_{3}^{{\varvec{C}}}=\frac{1}{2} {{\varvec{V}}}_{4}^{{\varvec{D}}} \Rightarrow \Vert {{\varvec{V}}}_{3}^{{\varvec{C}}}\Vert =\frac{1}{2} \Vert {{\varvec{V}}}_{4}^{{\varvec{D}}}\Vert$$

28 $$\mathrm{If \,we\, pose} \Vert {{\varvec{V}}}_{4}^{{\varvec{D}}}\Vert =\Vert {\dot{y}}_{4}^{D}\Vert , \mathrm{we\, get} : \Vert {\dot{y}}_{4}^{D}\Vert =\frac{1}{2} \Vert {{\varvec{V}}}_{3}^{{\varvec{C}}}\Vert$$

As the acceleration is a non-equi-projective field, it is deduced from the temporal derivation of the field velocity.

## Results and discussion

As with the fixed compression ratio engine, the GBCM engine also has input–output relationships in position, speed, and acceleration between the rotation of the crank 1 and the vertical displacement of the piston 4. This mechanism also has only one useful mobility ${\theta }_{1}$.

**Figure 3:**
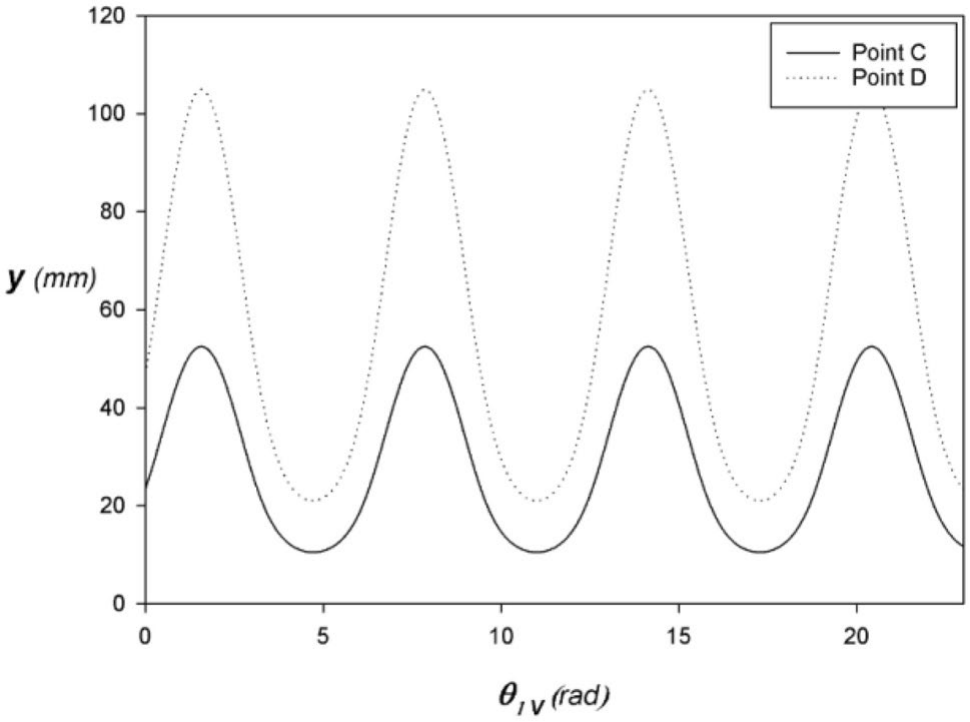
Position at C and D vs. angular displacement of the VCR engine with $\lambda =1/1.5$ and $N=3000 \,rpm$.

**Figure 4:**
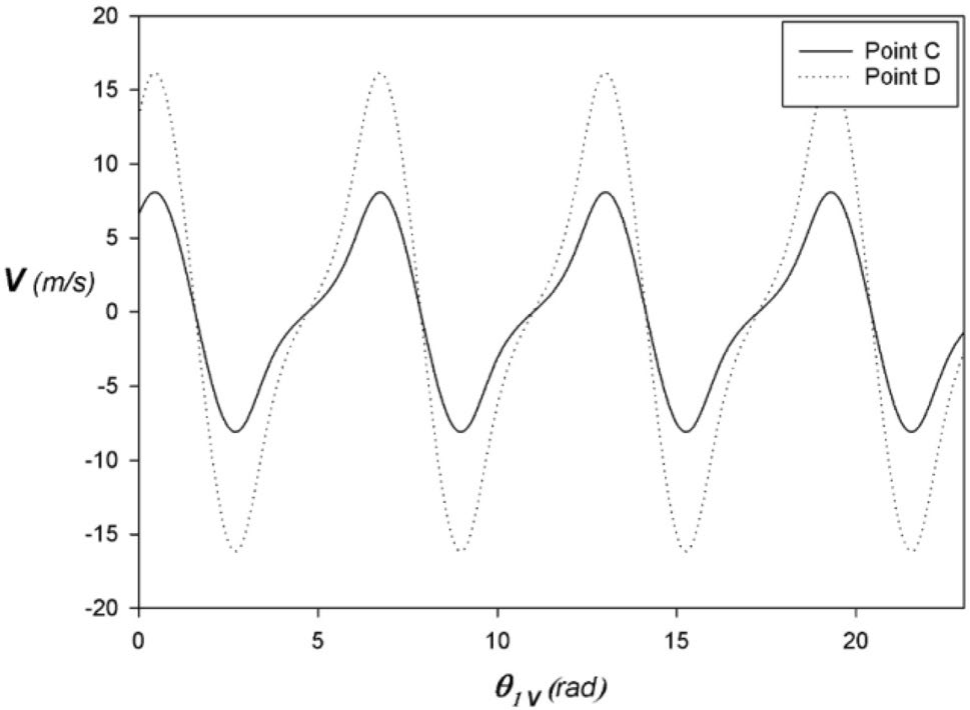
Speed at C and D vs. angular displacement of the VCR engine with $\lambda =1/1.5$ and $N=3000 \,rpm$.

**Figure 5:**
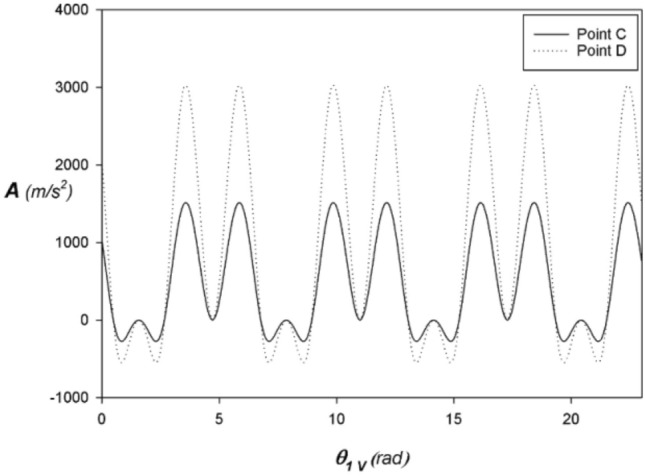
Acceleration at C and D vs. angular displacement of the GBCM engine with $\lambda =1/1.5$ and $N=3000\, rpm$.

Figures 3, 4, and 5 shows the alternative rectilinear position, velocity, and acceleration at points C and D respectively expressed as a function of the angle ${\theta }_{1}$ of the VCR engine crankshaft with $\lambda =1/1.5$. Thus, the kinematic quantities at point C are one-half of those at point D. As a result, only half the mass of the connecting rod is taken into account when calculating the inertial forces, giving the connecting rod increased structural rigidity to resist buckling.

In the steady-state $\theta =\omega t \Rightarrow \dot{\theta }=\omega$ with $\omega =2 \pi N/60$. If we assume an average engine speed of $N=3000\mathrm{\, rpm}$ we obtain $\omega =100 \pi rad/s$. Similarly, we pose ${L}_{1}=21 \,mm$ et $\lambda =1/1.5$.

To obtain a unit displacement of 1484/4 cm3 with a bore of 75 mm in diameter, it is necessary to travel a stroke of (2*L1 = 2*21 mm). With regard to semi-fast engines, the use of a corresponding lambda factor equal to 1/1.5 is often recommended. This choice is based on several technical considerations. It aims to reduce mechanical constraints, improve the durability of the engine and optimize its energy efficiency. This ratio between the stroke and the length of the connecting rod makes it possible to achieve a balance between engine speed and torque, thus ensuring optimal performance in different usage conditions.

Thus, the piston kinematics is strictly identical to that of a conventional engine with the same crankshaft ratio $\lambda$ regardless of the compression ratio and any gear wheel radius.

The stroke of the connecting rod head 2 at C is twice the crank radius $\mathcal{C}=2 {L}_{1}$ like the classic fixed compression ratio engine according to Figs. 3, 4, and 5 which show respectively the behavior of the reciprocating rectilinear position, velocity, and acceleration of the connecting rod heads 3 at point C expressed as a function of the angle ${\theta }_{1}$ of the crankshaft with ${\lambda }_{V}=1/1.5$ in a steady-state fixed at $N=3000 \,rpm$.

In the steady-state $\theta =\omega t \Rightarrow \dot{\theta }=\omega$ with $\omega =2 \pi N/60$. If an average engine speed is considered at $N=3000 \,rpm$ we obtain $\omega =100 \pi rad/s$ and we assume that ${L}_{1}=21 \,mm$.

With zero initial condition ${t}_{0}=0$ we will have ${\theta }_{1}=0$ therefore:

29 $$\overline{{\dot{y}}_{4}^{D}}= 2 N\mathcal{C}/60$$

The stroke is $\mathcal{C}=84 {10}^{-3}m$, then we get $\overline{{\dot{y}}_{4}^{D}}=8.4\, m/sec$ which means the average piston speeds for a given engine rpm. Manufacturers limit the maximum piston speed for reasons of the mechanical strength of the engine to around $20 \,m/sec$.

To make a turn, the connecting rod must be longer than the crank: ${L}_{2}>{L}_{1}$.

The middle of the stroke is not the same as ${\theta }_{1}=0$.

There are two points where the piston velocity at point D cancels to change the sign in ${\theta }_{1}=\pi /2$ and ${\theta }_{1}=3\pi /2$ whose maximums occur in these angular positions but not exactly, the offset is greater the shorter the connecting rod length ${L}_{2}$.

The length ${L}_{2}$ of the connecting rod does not affect the piston stroke.

When ${L}_{2}$ is large enough, the acceleration becomes a quasi-periodic function.

## Conclusion

The kinematic modeling allowed us to simulate kinematic and geometric parameters for the gear-based crank mechanism engine.

The study has highlighted that the kinematic quantities at connecting rod head are one-half of those at the piston top head. It follows that only half the mass of the connecting rod is taken into account when calculating the inertia forces.

The stroke of the connecting rod head is twice the crank radius like the classic fixed compression ratio engine.

Thus, all the extrinsic kinematic properties of a classic crankshaft mechanism of the fixed compression ratio engine remain valid for a gear-based crank mechanism engine, regardless of the gear wheel radius, the crankshaft ratio, the compression ratio i.e. the compression ratio adjustment position.

Finally, kinematic parameters such as piston stroke, positions, velocities, and accelerations, can be used for dynamic calculation purposes in terms of inertial properties, reactions, torques, and powers.

## Data Availability

All the data generated and analysed during this study are included in this article.

## Abbreviations

$${L}_{1}$$: Crank radius
${L}_{2}$: Connecting rod length
$\lambda$: Crank radius to connecting rod length ratio
$\mathcal{C}$: Engine piston stroke
$y , \dot{y} and \ddot{y}$: Displacement, velocity, and acceleration
${\theta }_{1} , {\dot{\theta }}_{1} and {\ddot{\theta }}_{1}$: Displacement, velocity, and angular acceleration of the crank
${{\varvec{V}}}_{{\varvec{n}}}^{{\varvec{M}}}$: Velocity vector of an M point of the n-th link
${{\varvec{A}}}_{{\varvec{n}}}^{{\varvec{M}}}$: Acceleration vector of an M point of the n-th link
${\theta }_{3}$: Angular displacement of gear wheel 3 in variable compression ratio configuration
${R}_{3}$: Radius of gear wheel 3 in variable compression ratio configuration
